# Task sharing in elective inguinal hernia surgery in Ghana: a workforce model comparing surgeons and physicians

**DOI:** 10.1093/bjs/znaf173

**Published:** 2025-09-04

**Authors:** Mwayi Kachapila, Stephen Tabiri, Mark Monahan, Francis A Abantanga, Anita Eseenam Agbeko, Fareeda Agyei, Aneel Bhangu, Dion G Morton, Tracy E Roberts, Virginia Ledda, Mike Ohene-Yeboah, Raymond Oppong

**Affiliations:** NIHR Global Health Research Unit on Global Surgery, School of Health Sciences, College of Medicine and Health, University of Birmingham, Birmingham, UK; School of Health Sciences, College of Medicine and Health, University of Birmingham, Birmingham, UK; Global Health Research Unit on Global Surgery, Ghana Research Hub, Tamale, Ghana; School of Medicine, University for Development Studies and the Tamale Teaching Hospital, Tamale, Ghana; Ghana Hernia Society, Accra, Ghana; School of Health Sciences, College of Medicine and Health, University of Birmingham, Birmingham, UK; School of Medicine, University for Development Studies and the Tamale Teaching Hospital, Tamale, Ghana; Ghana Hernia Society, Accra, Ghana; Global Health Research Unit on Global Surgery, Ghana Research Hub, Tamale, Ghana; Department of Surgery, Korle Bu Teaching Hospital and University of Ghana Medical School, Accra, Ghana; Global Health Research Unit on Global Surgery, Ghana Research Hub, Tamale, Ghana; Directorate of Surgery, Komfo Anokye Teaching Hospital, Kumasi, Ghana; NIHR Global Health Research Unit on Global Surgery, School of Health Sciences, College of Medicine and Health, University of Birmingham, Birmingham, UK; School of Health Sciences, College of Medicine and Health, University of Birmingham, Birmingham, UK; NIHR Global Health Research Unit on Global Surgery, School of Health Sciences, College of Medicine and Health, University of Birmingham, Birmingham, UK; School of Health Sciences, College of Medicine and Health, University of Birmingham, Birmingham, UK; School of Health Sciences, College of Medicine and Health, University of Birmingham, Birmingham, UK; NIHR Global Health Research Unit on Global Surgery, School of Health Sciences, College of Medicine and Health, University of Birmingham, Birmingham, UK; School of Health Sciences, College of Medicine and Health, University of Birmingham, Birmingham, UK; Ghana Hernia Society, Accra, Ghana; Department of Surgery, Korle Bu Teaching Hospital and University of Ghana Medical School, Accra, Ghana; NIHR Global Health Research Unit on Global Surgery, School of Health Sciences, College of Medicine and Health, University of Birmingham, Birmingham, UK; School of Health Sciences, College of Medicine and Health, University of Birmingham, Birmingham, UK

Over one million inguinal hernias were untreated in Ghana between 2012 and 2022 due to a shortage of surgeons and a lack of surgical care close to patients’ communities^1,2^. Task sharing is a process whereby specific roles are moved from one cadre of the workforce to another; however, both cadres provide the service to maximize population benefits. This model has shown potential for expanding access to safe surgical care in low-resource settings^3^. Task sharing between surgeons and non-surgeon physicians for inguinal hernia repair is being explored in Ghana to reduce unmet need^2,4^. An RCT is soon to be launched across 18 district hospitals in Ghana to assess the safety and clinical impact of this change in practice.

In anticipation of this study, a decision-analytic model was built using published data and informed by site visits to five district hospitals. A healthcare provider perspective was adopted, and costs were estimated using a top-down approach. It was assumed that non-surgeon physicians would conduct hernia repairs for up to six years after completion of training, and anticipated volume was estimated from the data available. The cost of a hernia repair was taken from a published study comparing hernia repair undertaken by non-surgeon physicians and surgeons^5^. Resources for training one surgeon were calculated to equate to the resources for training 12 non-surgeon physicians at simple hernia repair (both for time and cost). The analysis estimated the cumulative number of hernia repairs performed over time by 12 non-surgeon physicians compared with one surgeon, alongside the costs of the surgery performed. The reported costs include the average cost of surgical training (€14 720), the average cost of training non-surgeon physicians (€1 225), the cost of hernia repair, and cost of recurrence and reoperation.

After 6 years of repairing hernias the 12 non-surgeon physicians performed 4625 hernia repairs at a cost of €624 543(Euros), compared with 784 hernia repairs performed by a surgeon at a cost of € 109,921. As such, the non-surgeon physicians performed 3841 more hernia repairs at an additional cost of € 514,622 (*Fig. 1*). The cost per hernia repair, inclusive of training, was comparable (non-surgeon physicians €135, surgeon €140). In a sensitivity analysis, even if the dropout rate of non-surgeon physicians reaches 100% between 3 and 6 years, the number of hernia repairs completed will still be almost 4-fold that achieved by the surgeon and the per-procedure costs remain comparable (*Fig. 1*).

**Fig. 1:**
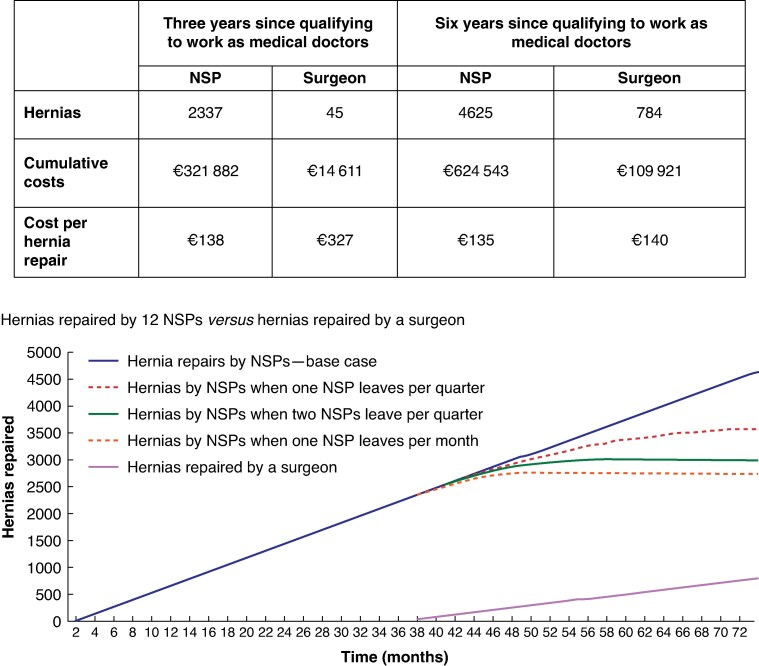
Hernia repairs performed by 12 non-surgeon physicians (NSPs) and a surgeon, and associated costs

Expanding training for surgeons in Ghana cannot address the current unmet need for inguinal hernia repair; however, short- and medium-term access can be improved by training non-surgeon physicians with comparable procedure costs. Non-surgeon physicians are not a replacement for surgeons but can be cost-effective in meeting medium-term capacity needs for specific procedures in a task-sharing capacity. One limitation of this analysis is that it did not consider the productivity gains associated with increasing access to surgery.

Expanding access to hernia repair will require more resources including operating theatres and consumables. The next step is to evaluate the clinical and patient benefits of such a development, and the impact this model may have beyond the borders of Ghana.

## Data Availability

The data set used in this study is not publicly accessible but can be made available when a reasonable data request has been made to the authors.
